# A Case

**Published:** 1884-11

**Authors:** 


### A Case.

The following, from Dr. J. W. Baxter, Jr., of Vevay, Ind., is well worthy of record, and will be of interest to every dentist who may have met with similar difficult cases:

He remarks that on March 27th last, Mr. M——, aged twenty-five, of nervous, sanguine temperament, called to have two teeth extracted—the second bicuspid and first molar, lower jaw, left side. After making a careful examination, the patient was presuaded to have them filled, preparatory to which, the rubber dam was adjusted and both cavities excavated, in doing which, however, the pulps were exposed. The hemorrhage occasioned some delay, after which the cavities were dried and treated with carbolic acid; dried, and filled with phosphate of zinc. The gentleman exhibited signs of general depression and feebleness, evidently from malarial influence. Elixir of iron, quinine and strychnia was prescribed, prepared by Diehl, of Louisville, Ky.

The teeth continued painful, and he called the next day to insist that both teeth be extracted, as he was suffering intense pain in the region of the left ear. He was induced to postpone the extraction, for further investigation of the case with a view of ascertain if the pain was not kept up by difficulty at some other point. An impression of the teeth of both jaws was taken to note particularly the arrangement of the occlusion, upon which it was found that the upper, third molar was crowded out of the arch, to the extent of two-thirds of its width, the inner cusps striking the lower, third molar in such a way as to press it forcibly backward, and to make undue pressure upon the parts influenced by this force. This caused the tooth to change its position when the jaws were firmly closed. This difficulty being fully recognized, it was decided to remove the upper, third molar, which was done, after which the pain gradually subsided, recurring only occasionally, three or four times, for a brief period. The prescription already mentioned was now resorted to, and with the most satisfactory results.

After the removal of the third molar, it was found that an abscess of rather chronic character was being formed in the bifurcation of the roots. The teeth that were filled seemed thus far to be right.

The especial interest that attaches to this case rests in the fact of the remote point of irritation that, exciting what seemed to be, by reflex influence, pain in the bicusped and first molar in the inferior jaw. It emphasizes the importance of the thorough examination of all parts of the mouth in every case that seems obscure, and is not perfectly plain upon a first examination. Such cases call for a thorough comprehension of reflex nervous influences, and of peculiar conditions that are liable to exist in connection with diseased teeth.

